# Right hemisphere involvement in intensive aphasia therapy outcomes: a multimodal neuroimaging concept study

**DOI:** 10.1007/s00415-026-13999-4

**Published:** 2026-07-24

**Authors:** Nina Scholtes, Stefan Heim, Ferdinand Binkofski, Cornelius J. Werner

**Affiliations:** 1 Department of Neurology, Medical Faculty, RWTH Aachen University, Aachen, Germany; 2 Division of Neuropediatrics and Integrated Health Care, Department of Pediatrics and Adolescent Medicine, KJF Klinikum Josefinum, Augsburg, Germany; 3 Department of Psychiatry, Psychotherapy and Psychosomatics, Medical Faculty, RWTH Aachen University, Aachen, Germany; 4 Institute of Neuroscience and Medicine (INM-1), Research Centre Jülich GmbH, Jülich, Germany; 5 Institute for Midwifery, Medical Faculty, RWTH Aachen University, Aachen, Germany; 6 Division for Clinical Cognitive Sciences, Department of Neurology, Medical Faculty, RWTH Aachen University, Aachen, Germany; 7 Institute of Neuroscience and Medicine (INM-4), Research Centre Jülich GmbH, Jülich, Germany; 8 Department of Neurology and Geriatrics, Johanniter Hospital, Stendal, Germany

**Keywords:** Aphasia, Intensive language therapy, Neuroplasticity, Structural MRI, Functional MRI, Lesion volume

## Abstract

**Background:**

Few studies investigated factors that predict intensive language therapy (ILT) outcome. This study aimed to analyze multimodal neural factors that possibly underly ILT success.

**Methods:**

Lesion volume and DTI analyses were performed with 17 aphasic individuals, and complemented by fMRI analysis in 12 patients (due to drop-outs). Structural and functional data were collected at the beginning of a seven week ILT program and correlated with language changes, measured using the Aachen Aphasia Test. Additionally, all analyses were correlated with the patients’ initial aphasia severity.

**Results:**

Therapy success was associated with functional activity in right inferior and middle temporal gyri, bilateral superior frontal gyrus/ adjacent anterior cingulate cortex, and right occipital lobe (lingual gyrus, fusiform gyrus, lateral occipital cortex). Statistically uncorrected DTI analysis (*p*(uncorr) < .001) revealed a positive correlation between language improvement and structural integrity in the right temporal lobe and two left-hemisphere white matter clusters (frontal, parietal). Neither global nor language-specific lesion volume was correlated with therapy outcome. However, lesion volume in insula, putamen and white matter tracts was correlated with initial aphasia severity.

**Conclusions:**

Our preliminary results suggest a predictive role of right temporal structures and functional activation in areas associated with healthy language processing. They also indicate a contribution of functional activation in brain areas associated with cognitive control and non-linguistic semantic processing. Further, lesion volume in both global and language-specific structures proved relevant for the initial severity of aphasia but not for recovery potential following ILT. Our results should be regarded as hypothesis-generating for future research.

## Introduction

Aphasia is a language disorder that roughly affects one third of stroke survivors, often leading to debilitating limitations in everyday life [1]. Intensive language therapy (ILT) can significantly reduce symptom severity, even in the chronic stage (> 12 months post-onset) [2]. However, not all patients profit from therapy [1]. Research has focused on identifying the neural correlates of mechanisms relevant for language recovery, primarily examining the factors influencing spontaneous recovery or neuroplastic changes induced by aphasia therapy. However, the underlying mechanisms to a positive treatment response remain elusive. To establish the backdrop to our study, we will briefly discuss current literature regarding spontaneous recovery and treatment-induced structural and functional changes, before summarizing evidence of predictors to therapy success.

### Structural influence factors to spontaneous and therapy-induced aphasia recovery

Global lesion size has been proposed as a key factor affecting both the trajectory of spontaneous language recovery [3] and overall aphasia severity [4]. However, lesion volume in specific language-related regions—such as the inferior and middle frontal gyrus (IFG; MFG), supramarginal gyrus, and angular gyrus—may be more predictive of aphasic symptom severity than global lesion size [4,5]. Similarly, subcortical structures, including the insula (INS), thalamus (THA), and basal ganglia—particularly the putamen (PUT) and caudate nucleus (CAU)—have been linked to specific aphasic symptoms and their severity [6,7].

Regarding white matter structures, some studies have shown a link between the degree of spontaneous recovery and the extent of damage in the left uncinate fasciculus (UF) and the superior and inferior longitudinal fasciculi (SLF, ILF) [8]. In terms of white matter plasticity, studies reported therapy-induced changes in the structural integrity of the left ILF [9], as well as increased volume in left [10] and right arcuate fasciculus (AF) [11], which was correlated with positive treatment effects especially in connection with intensive, specialized treatment protocols like melodic intonation therapy [11].

### Functional influence factors to spontaneous and therapy-induced aphasia recovery

Functional imaging studies have demonstrated a relationship between left hemisphere activity and overall language improvement. There is some consensus that activity in areas that are associated with language processing in healthy participants, such as the left IFG, plays a crucial role in language recovery [12], and it has been proposed, that optimal recovery relies on the normalization of activity in language related left-hemisphere areas [13].

The influence of right hemisphere activity, however, remains highly debated. Some studies have linked activity in the right IFG, middle temporal gyrus (MTG), precentral gyrus, and INS to successful spontaneous or treatment-induced language recovery [14,15], whereas others found no significant impact or even a negative correlation between right hemisphere activity and the course of language recovery [13, 16]. The latter has been attributed to maladaptive compensatory processes or transcallosal inhibition [17]. However, inhibiting right hemisphere activity—e.g., in the IFG—through transcranial magnetic stimulation, while pointing towards positive effects overall, has not consistently led to improved language performance for all patients [18]. In addition, studies have demonstrated that functional activity during the course of recovery is not necessarily limited to one hemisphere, for example, with findings showing the recruitment of bilateral frontal brain regions associated with anomia treatment [19]. Saur et al.  [20] described both increased contralesional and decreased ipsilesional activity acutely after stroke, followed by upregulation in unlesioned left hemispheric areas as well as language homologous right hemisphere areas, especially in Broca’s homologue, two weeks post-onset. This was subsequently followed by a decrease in right hemispheric activity in favor of a normalization of left hemispheric activity after one year.These results may, however, not be readily transferred to therapy-induced language improvement [1].

Additionally, several brain regions exhibit bilateral activity during healthy language processing, including the superior temporal gyrus (STG) and sulcus (STS), posterior MTG, and inferior temporal gyrus (ITG) [21,22]. This is thought to be a remnant of early language development, during which initially bilaterally located language network areas become increasingly left-lateralized, leaving potential for right-hemisphere activity into adulthood [23]. Thus, right hemisphere activity in these areas may also reflect a patient’s capacity to recruit intact structures in bilateral regions in healthy speakers.

### Structural and functional predictors to aphasia therapy success

Research examining predictors of (intensive) language therapy success through the prediction of therapy outcome from structural or functional baseline measures has been relatively scarce [24,25].

Regarding structural factors, as lesion location and extent limit the capacity for neuroplasticity [25], an influence of lesion characteristics to therapeutic outcomes is generally assumed [24]. Current findings suggest, however, that global lesion size does not influence treatment response [26,27], while some studies point towards a negative impact of specific damage in the left MTG and temporoparietal junction to therapy success [24]. Regarding white matter, in our previous work, we found no correlation between lesion volume in AF and SLF and the success of intensive aphasia therapy in four exemplary cases [26]. However, other studies proposed that structural integrity in the left ILF [9] and overall structural integrity in the left temporal lobe [28] may be predictive of positive treatment outcomes. Thus, therapy success might depend on intact structural connectivity, yet growing evidence suggests that therapy success is not solely dependent on lesion characteristics.

Regarding functional activity, results from one study suggest that pre-treatment functional activity in the remaining parts of the language network within the perilesional frontal lobe may be associated with therapy success [29]. Others reported, that anomia therapy success was predicted by baseline activity in the left precentral gyrus [30]. In addition, further research has shown pre-treatment right-hemisphere functional activation in lesion-homologue areas, such as the right IFG [15], and subcortical functional activation, particularly in the left-hemispheric caudate nucleus [31], to be relevant for therapy success. Current literature suggests that positive treatment response might be driven by a combination of multiple processes, possibly including both mechanisms of structural neuroplasticity and functional reorganization [24].

As yet, this line of research remains underrepresented, although the available evidence indicates that pre-treatment structural characteristics and functional activity may hold ‘potential for predicting therapeutic outcomes’ [25]**.** Different symptoms or lesion characteristics may necessitate the application of different treatment methods to offer the best therapy approach for an individual patient [25]. To achieve this, however, broader knowledge of the factors that underly therapy success is needed.

So far, in imaging studies concerning aphasia recovery, language ability and therapy outcomes were often assessed in relation to specific language modalities, such as naming [14], and were frequently investigated at the single-word level [32]. While this approach provides valuable insights into the recovery of specific language modalities, it does not fully capture the variability of aphasic symptoms and recovery. Additionally, fMRI designs and aphasia treatment contents often targeted on the same functions, therefore effects of fMRI task and therapy method might be intertwined [32]. To address these points, we assessed language performance across multiple linguistic levels to better capture the variability of aphasic symptoms and individual treatment responses, rather than focusing on a single language modality. In addition, we chose to separate fMRI paradigm task content from the content of the individual ILT.

This study was conducted to identify factors that possibly underly intensive aphasia therapy success. Given the limited research in this area, we employed multimodal imaging methods, including both structural and functional imaging, in an exploratory study design.

### Study aims

This study aimed to investigate potential pre-treatment predictors to ILT success regarding (1) structural influence factors and (2) functional brain activation in stroke-induced aphasia. Concerning (1), in line with the literature as previously described, we hypothesized that structural factors of possible relevance to ILT success might comprise both lesion volume and/or structural white matter integrity. Therefore, we addressed the following research questions: Is therapy success correlated with (i) lesion volume in global and/or language specific areas? Is there an association between (ii) pre-treatment structural integrity of language-specific white matter tracts with ILT success? Regarding aim (2), research questions were: Is there an association between (i) functional activation pre-ILT and therapy success? If so, are effects associated with (ii) left- or right hemisphere activation; and/or (iii) with functional activity in language-specific brain areas?

## Materials and methods

### Participants

Patients were recruited from the Aphasia Rehabilitation Ward of the Department of Neurology at RWTH Aachen University Hospital, where they stayed—independently of this study—to participate in a specialized ILT program. All patients met the following inclusion criteria:

1. Clinically diagnosed aphasia with sufficient language comprehension to follow study instructions,
2. MRI compatibility,
3. Monolingual German speaker,
4. Right-handedness, as determined by the Edinburgh Handedness Inventory (laterality index ≥ 80) [33],
5. Single left-hemisphere stroke,
6. Post-acute or chronic recovery phase (≥ six weeks post-onset),
7. No additional neurological or psychiatric disorders.

Patient characteristics and therapy outcomes are presented in Table 1. Initially, 17 patients were recruited. However, due to artifacts (motion; growing pain while lying still (two patients each)) and one case of computer malfunction (by not successfully playing the auditory stimuli), functional imaging data could only be analyzed for a subgroup of 12 participants. Even though a larger sample size would have been preferable, we consider this number sufficient to generate hypothesis-driven insights, consistent with the preliminary and exploratory nature of our study.

**Table 1:** Patient characteristics and therapy outcomes

Patient No	Age (5 y-interval)	Time p.o. (years; months)	Language Ability (AAT)					
			Pre-test scores (t-values)	ΔAAT	Aphasia severity	Lesion Volume (mm^3^)	Lesion location** (covered brain areas within lesion)	
							Cortical	Subcortical
1	51–55	0;9	41.9	1.11	severe	158 600	Fro (IFG to Central Sulcus)	INS, PUT
2	51–55	0;11	40.5	1.03	severe	239 240	Fro, Temp, Par (IFG to MTG and postcentral Gyrus)	INS, PUT
3	46–50	0;8	50.1	–1.62	moderate	121 360	Par (parietal Operculum to IPL)	INS
4	51–55	1;5	57.9	0.73	mild	147 408	Fro, Temp, Par (IFG to STG and IPL)	INS, PUT
5	51–55	0;11	40.9	0.87	severe	286 960	Fro, Temp, Par, Occ (left hemisphere from IFG to STG and LOC/FUS)	INS, PUT, THA
6	66–70	2;7	43	–3.16°	severe	349 280	Fro, Temp, Par (left hemisphere from IFG/frontal Operculum to STG and LOC/FUS)	INS, PUT
7	46–50	0;5	46.2	4.7*	moderate	164 104	Fro, Temp, Par (frontal and parietal Operculum, STG)	INS, PUT
8	21–25	4;3	48.9	1.27	moderate	393 896	Fro, Temp, Par, Occ (MFG, IFG to STG, IPL and LOC)	INS, PUT
9	56–60	3;8	47.9	0.41	moderate	168 248	Fro, Temp, Par (MFG, IFG to STG, MTG and parietal Operculum)	INS, PUT
10	41–45	0;6	72.5	0.67	mild	106 400	Fro, Par (SFG to postcentral Gyrus)	–
11	46–50	3;4	48.6	–1.76	moderate	406 832	Fro, Temp, Par, Occ (left hemisphere from SFG to ITG and LOC/FUS)	INS, PUT
12	46–50	1;6	43.7	0.17	severe	229 888	Fro, Temp, Par (IFG to MTG and IPL)	INS, PUT
13	46–50	0;4	50.7	8.26*	moderate	158 048	Fro, Temp, Par (frontal Operculum to MTG and IPL)	INS, PUT
14	21–25	0;5	47.3	3.37*	moderate	149 640	Fro, Temp, Par (frontal Operculum to MTG and IPL)	INS, PUT
15	36–40	0;4	57.1	–0.12	mild	262 920	Fro, Temp, Par (MFG to STG and IPL)	INS, PUT
16	46–50	5;1	59.9	–3.0°	mild	116 480	Fro, Temp, Par (frontal Operculum to STG and IPL)	INS
17	61–65	0;2	53.6	1.91*	moderate	87 488	Fro, Par (IFG to parietal Operculum)	INS, PUT
Mean (SD)	48;9	1;6	50.04	0.87 (2.77)		208 634.82		
range	23;4 – 66;4	0;2 – 5;1	40.5 – 72.5	–3.16 – 8.26	n = 17 mild: n = 4 moderate: n = 8 severe: n = 5	87 488 – 406 832		

*significant improvement (*p* < .1), °significant deterioration (*p* < .1), evaluated with the psychometric single case diagnostics, automatized by computer program AATP [39]

*SD*   standard deviation, *Fro*   frontal lobe, *Temp*   temporal lobe, *Par*   parietal lobe, *Occ*   occipital lobe, *S/ M/ IFG*   superior/ middle/ inferior frontal gyrus, *S/ M/ ITG*   superior/ middle/ inferior temporal gyrus, *IPL*   inferior parietal lobe, *LOC*   lateral occipital cortex, *FUS*  fusiform gyrus, *INS*  insula, *PUT*   putamen, *THA*   thalamus

**The anatomical structure is reported, if > 10% of that structure is affected by the lesion. Lesion description following the Ebrains Julich-Brain Atlas [66]

### Design

During the first week of a seven week in-patient stay at our facility, pre-testing of language abilities with the highly robust and validated German Aachen Aphasia Test (AAT) [34] as well as the execution of all structural (T1, FLAIR, DTI) and functional MRI scans, was conducted. Following pre-testing, patients participated in the Aphasia Rehabilitation Ward’s standardized ILT program, where at least 10 h of language therapy per week in individual and group sessions (min. 2 h/day, five days/week; at least 5 h individual therapy) [35,36] are provided. Evidence-based treatment aims and procedures were functionally tailored to each patient’s individual aphasic symptoms and deficits, as determined by their pre-test results in language testing. For individual therapy, these aims and procedures were chosen by each patient’s main therapist at the beginning of the treatment program, whereas group sessions, consisting of 4–5 patients per group, targeted deficits that were relevant to all patients in the same group (e.g. language comprehension tasks or naming tasks). This treatment approach has long been established as an effective therapy procedure and is published elsewhere [35, 37].

In the seventh week, testing of aphasic language abilities with the AAT was repeated to evaluate individual treatment success and to subsequently examine a possible correlation between therapy outcomes and pre-therapy structural/functional factors. ILT, as well as study-relevant AAT testing before and after therapy, was conducted by different personnel to ensure objective results. However, each person involved in aphasia testing and treatment was a Speech and Language Pathologist specialized in aphasiology. In line with our inclusion criteria, our patient group showed wide differences in illness duration (Table 1). Therefore, the influence of spontaneous recovery was controlled as covariate in every analysis we conducted.

### Neuroimaging

#### fMRI task

All participants performed a block design fMRI auditory language processing task with auditory stimuli from four different stimulus groups:

- 1. Strings of meaningful words,
- 2. Strings of pseudowords,
- 3. Meaningful sentences,
- 4. Pseudosentences.

**Fig. 1:**
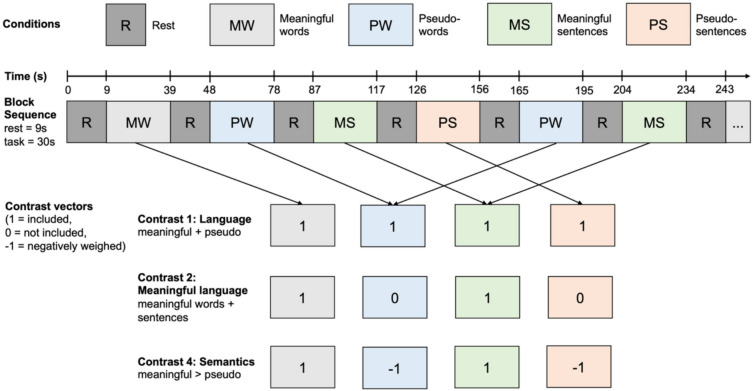
Depiction of an exemplary fMRI paradigm presentation including conditions, timeline and block sequence as well as examples of the contrast vectors/ contrasts of interest. Stimulus blocks were presented in a randomized order to each patient. Contrast 3 (Pseudo Language, contrast vector ‘0 1 0 1’), consisting of an additive combination of pseudowords and -sentences, was structured analogously to Contrast 2, and has therefore been excluded from this figure due to space constraints

Each stimulus group consisted of three different stimulus sets, therefore resulting in 12 blocks per run, which were presented in a randomized order to each patient. Each block presentation lasted 30 s, followed by a 9 s pause (Fig. 1). These stimuli were previously used in another study [38] and were therefore validated as suitable for examining language-related fMRI activity. Patients were instructed to listen to the auditory stimuli. A language production task was not included, to minimize motion artefacts and to ensure that even severely impacted patients could perform the task. Stimuli were presented using the computer program “Presentation” (Neurobehavioral Systems, Albany, USA).

#### Data acquisition

All MRI scans were acquired on a 3.0 T Philips scanner (Philips Healthcare) using an 8-canal phased-array head coil. Structural scans were obtained on these parameters:

T1: Repetition time/echo time: 9.9/4.6 ms; flip angle: 80°, field of view: 256 mm; acquisition matrix: 256 × 256; slice thickness: 1 mm, acquired voxel size 1.0 × 1.0 × 1.0 mm.

FLAIR: Repetition time/echo time: 11,000/125 ms; field of view: 224 mm; acquisition matrix: 312 × 157; slice thickness: 3 mm, acquired voxel size 0.72 × 1.13 × 3 mm.

DTI parameters were obtained using a diffusion-echo-planar-imaging-sequence with the following parameters: Repetition time/echo time: 7552/80 ms; flip angle: 90°, field of view: 224 mm; acquisition matrix: 112 × 110; slice thickness: 2 mm, acquired voxel size 2.0 × 2.0 × 2.0 mm; no slice gap; attenuation factor: b = 1000 s/mm^2^.

Functional MRI images were acquired with an echo-planar-imaging-sequence with these parameters: Repetition time/echo time: 3000/30 ms; flip angle: 90°, field of view: 224 mm; acquisition matrix: 80 × 80; slice thickness: 3 mm, slice gap: 0.5 mm, acquired voxel size 3.0 × 3.0 × 3.0 mm.

### Data analysis

#### Behavioral testing

AAT mean profile height (MPH) was calculated for each patient at the two time points (t1: before, t2: after therapy) using the computer program ‘Aachener Aphasietest Programm’ (AATP) [39]. Clinically relevant change at the individual level was determined by the psychometric single-case diagnosis [40] implemented in the AATP, which tests whether each patient's pre–post difference exceeds the measurement error of the AAT within its normative sample (Table 1). MPH was derived from the patients' scores on multiple linguistic AAT subtests (Token Test, Speech Repetition, Naming, Written Language, and Language Processing) and is a measure for aphasia severity (t-values). Since it is composited of the results in all AAT subtests, MPH is also sensitive to changes in language abilities. To evaluate group effects, a univariate ANCOVA with repeated measures (*p* < 0.05; covariate: time post-onset) was performed to examine whether the mean values of the dependent variable (AAT) deviate significantly from each other within the two time points (t1 and t2), while controlling for the duration of illness. In addition, partial correlations, again controlling for illness duration, were applied to examine a possible correlation between the amount of therapy hours and outcome success that may have affected our results. For all MRI analyses, therapy-induced changes in AAT performance (ΔAAT: MPH at t2 minus MPH at t1) were calculated and subsequently used as continuous regressor.

#### Lesion volume

Each patient’s individual lesion mask was hand drawn, registered to MNI152 standard space, and overlaid with the normalized anatomical mask for each structure of interest to determine the amount of lesion to the specific structure. For a detailed description, see [26]. Anatomical structures of interest were selected based on their relevance to both healthy language processing and aphasia symptom severity:

- Cortical structures: Frontal lobe, temporal lobe, parietal lobe, occipital lobe.
- Subcortical structures: INS, THA, PUT, CAU.
- White matter tracts: AF, SLF, ILF, UF, middle longitudinal fasciculus (MdLF), extreme capsule (EmC), inferior fronto-occipital fasciculus (IFOF).

Global lesion volume, as well as lesion volume in each (sub)cortical and white matter structure was correlated with ΔAAT using partial correlations (one-sided, *p* < 0.05; covariate: time post-onset).

#### DTI-data

A voxel-based whole-brain analysis of the patients’ fractional anisotropy (FA) was conducted, as FA is considered a reliable measure of white matter integrity, due to its dependency on factors such as the density and volume of myelinated axon fibers [41]. The analysis was performed using Tract-Based Spatial Statistics (TBSS) [42], and followed the standard TBSS preprocessing steps described by [42] to obtain individual FA images and the FA-average group skeleton. After acquiring these images, individual FA images were projected onto the FA skeleton using a FA threshold of 0.3 to exclude gray matter voxels. Data were analyzed at the group level using FSL’s randomise [43] with one-sample t-tests in a General Linear Model (GLM) framework, applying 10,000 permutations (dependent variable: ΔAAT, covariate: time post-onset)**.** The analysis was conducted using threshold-free cluster enhancement (TFCE). Due to the small sample size and exploratory nature of our study, the DTI analysis was performed primarily uncorrected (*p* < 0.001). Subsequently, correction for multiple comparisons was performed by means of TFCE-based permutation testing (10,000 permutations) in FSL randomise, controlling the Family-Wise Error Rate (FWER) at *p* < 0.05 to evaluate whether the obtained results survived statistical adjustment. In addition, a post-hoc sensitivity analysis was conducted using G*Power 3.1 [44] (point-biserial correlation model; one-tailed; alpha = 0.05; power = 0.80; *n* = 17) to estimate the minimum detectable effect size. Only clusters containing more than ten consecutive voxels were considered [45].

#### Functional data

The analysis was conducted with FSL Version 5.0 [46]. Preprocessing steps were performed using FSL FEAT (FMRI Expert Analysis Tool) and included head motion correction and spatial smoothing with a Gaussian kernel of 6 mm FWHM on the blood-oxygenation-level-dependencies (BOLD) response data. To compensate for the extensive lesions, individual lesion weights were integrated into the registration process. For this, lesion weights were obtained by inverting the binary numbers in a manually constructed lesion mask for each patient (i.e. a “zero” was assigned to each lesioned voxel), resulting in the registration process ignoring areas with zero-voxels within these masks. This led to a minimization of the lesion-induced distortion. Registration of fMRI images to anatomical data was performed using FSL FLIRT [47], followed by nonlinear registration to MNI152 space with FSL FNIRT [48]. To remove motion artifacts, Independent Component Analysis (ICA) was conducted using ICA-AROMA (ICA-based Automatic Removal of Motion Artifacts) [49]. The denoised data was then further processed by applying high-pass filtering to each contrast (“Language”: cutoff-point 100 s; all other contrasts: cutoff-point 180 s). Different cut-off points were applied to account for the varying time points of the stimulus block presentation: In the “Language” contrast, the duration between the beginning of each stimulus block presentation and pause was consistent, as all the stimulus blocks were considered in the analysis. For the other contrasts, cut-off points were matched to the longest interval between two similar stimulus blocks, as calculated with FSL tools (cutoffcalc).

In the First-Level Analysis, preprocessed data was submitted to a GLM. Onset vectors for each of the four linguistic conditions were defined separately (Fig. 1). At the first level, each condition was contrasted against rest to obtain contrast images for the second level analysis. The following contrasts of interest were defined:

- "Language" (additive combination of all variables, contrast vector ‘1 1 1 1’),
- "Meaningful Language" (additive combination of meaningful words and sentences, contrast vector ‘1 0 1 0’),
- "Pseudo Language" (additive combination of pseudowords and pseudosentences, contrast vector ‘0 1 0 1’),
- "Semantics" (differential contrast: Meaningful Language > Pseudo Language, contrast vector ‘1 -1 1 -1’).

At the Higher-Level Analysis, the contrast images derived from the First-Level statistical analyses were examined using one-sample t-tests (covariate: time post-onset). This approach assessed the mean functional group activity in relation to positive treatment outcomes (ΔAAT) as a continuous regressor. In doing so, it enabled the identification of clusters whose activity was modulated by clinical improvements in language performance. All statistical analyses were performed using cluster correction via FWER correction (Cluster-Level Threshold Z > 2.3 and corrected significance level *p* < 0.05) as implemented in FSL FEAT.

#### Analysis in relation to aphasia severity

To verify that the results were related to therapy success rather than pre-therapy aphasia severity, all analyses were repeated as control analyses. These analyses followed the same procedures as described previously, except that AAT-MPH at the pre-testing level (time point t1) was used as the dependent variable instead of the therapy-induced change in AAT performance.

## Results

### Behavioral testing

The patient group of this study received a mean therapy amount of 13.11 full hours of therapy sessions per week (hours of individual therapy (mean): 10.58, standard deviation (SD): 2.77; group therapy mean: 2.54, SD: 0.72). In a repeated-measures general linear model with time post-onset as covariate, AAT-MPH improved significantly from pre (95% confidence interval (CI) 44.8 – 54.3) to post treatment (95% CI 46.6 – 55.3; main effect of time: F(1,15) = 7.64, *p* = 0.014). The change over time interacted significantly with time post-onset (F(1,15) = 5.79, *p* = 0.029), indicating that the magnitude of improvement was associated with the interval since stroke onset, consistent with a contribution of spontaneous recovery, for which we controlled by including time post-onset as a covariate throughout. The covariate showed no significant main effect on overall performance (F(1,15) = 0.07, *p* = 0.799). Partial correlations showed no association between the amount of therapy hours and language change (*p* = 0.592).

### Lesion volume

**Fig. 2:**
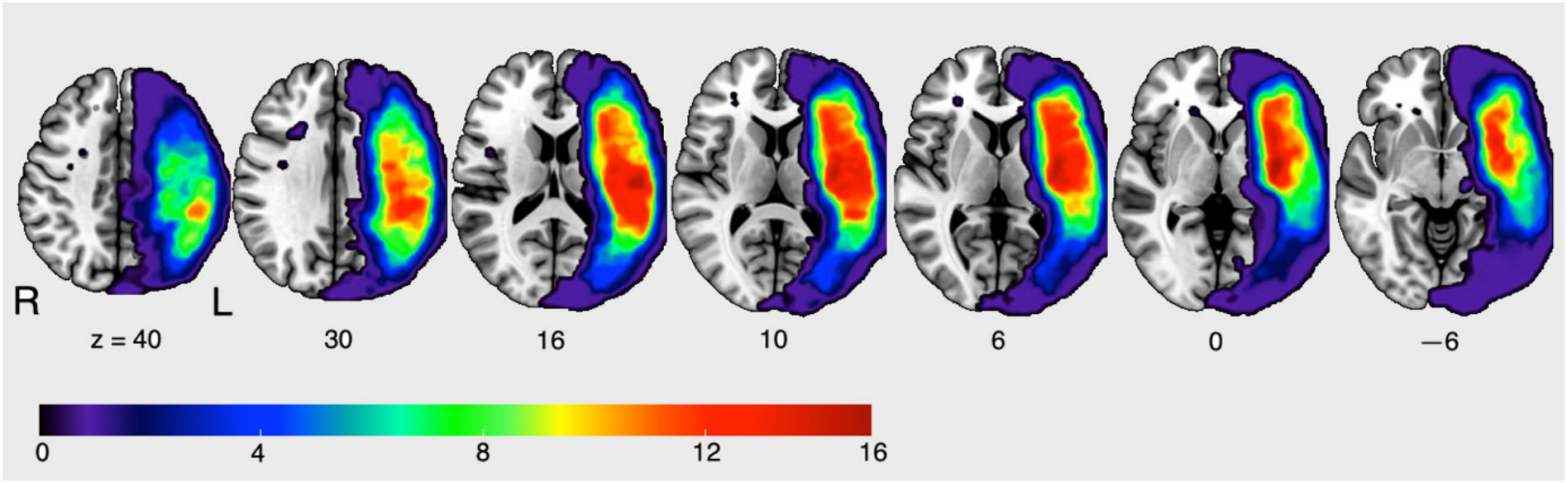
The lesion overlay map shows a maximal overlap of 16 lesions in left Rolandic Operculum, spreading into left INS, Planum temporale and Heschl’s Gyrus. The color bar indicates the number of overlapping lesions

The lesion overlay map showed a maximum overlap of 16 lesions, located in the Rolandic Operculum, spreading into the left INS, Planum temporale and Heschl’s Gyrus (Fig. 2). Lesion volume analyses revealed no significant correlation between either global lesion volume or lesion volume to specific areas with ILT success (Table 2).

**Table 2:** Correlation between ΔAAT / control analysis with damage to cortical, subcortical structures and white matter tracts

		Global lesion volume (mm^3^)	Cortical structures (% of damage to structures)			
			Fro	Temp	Par	Occ
	Mean damage	208 634.82	22.3	30.82	29.37	10.08
	range	87 488 – 406 832	0.88 – 68.8	0 – 93.25	1.97 – 80.2	0 – 76.59
ΔAAT	*r*-value	–0.149	–0.251	0.112	–0.244	–0.022
	*p*-value	0.582	0.348	0.689	0.363	0.936
Control testing	*r*-value	–0.33	–0.066	–0.428	–0.291	–0.247
	*p*-value	0.212	.809	.098	.273	.357

			Subcortical structures (% of damage to structures)			
			INS	PUT	THA	CAU
	Mean % damage		79.82	41.92	0.74	1.97
	range		0 – 100	0 – 84.65	0 – 12.0	0 – 9.7
ΔAAT	*r*-value		0.310	0.023	–0.136	–0.284
	*p*-value		0.243	0.932	0.615	0.286
Control testing	*r*-value		–0.752	–0.509	–0.276	–0.313
	*p*-value		< .001***	.044*	.301	.238

				White matter fiber tracts (% of damage to structures)				
		SLF	AF	ILF	MdLF	IFOF	UF	EmC
	Mean % damage	50.7	50.9	23.82	43.67	27.52	38.06	51.28
	range	15.19 – 92.59	15.22 – 91.68	0 – 85.67	0.52 – 88.31	0 – 79.58	0.02 – 86.19	0.22 – 96.09
ΔAAT	*r*-value	–0.254	–0.215	0.027	–0.044	–0.036	–0.092	–0.061
	*p*-value	0.3	0.424	0.922	0.871	0.894	0.736	0.822
Control testing	*r*-value	–0.650	–0.685	–0.440	–0.539	–0.529	–0.493	–0.725
	*p*-value	.006**	.003**	.088	.031*	.035*	.053	.001**

*significant correlation with *p* < .05, **p < .01, ***p < .001; partial correlations (controlled for covariate: time p.o.)

*Fro*   frontal lobe, *Temp*  temporal lobe, *Par*   parietal lobe, *Occ*   occipital lobe, *Ins*   insula, *Put*   putamen, *Tha*   thalamus, *Cau*   caudate nucleus;

*SLF/ILF*  superior/inferior longitudinal fasciculus, *AF*   arcuate fasciculus, *MdLF*  middle longitudinal fasciculus, *IFOF*   inferior fronto-occipital fasciculus, *UF*  uncinate fasciculus, *EmC*  extreme capsule

### DTI-analysis

**Fig. 3:**
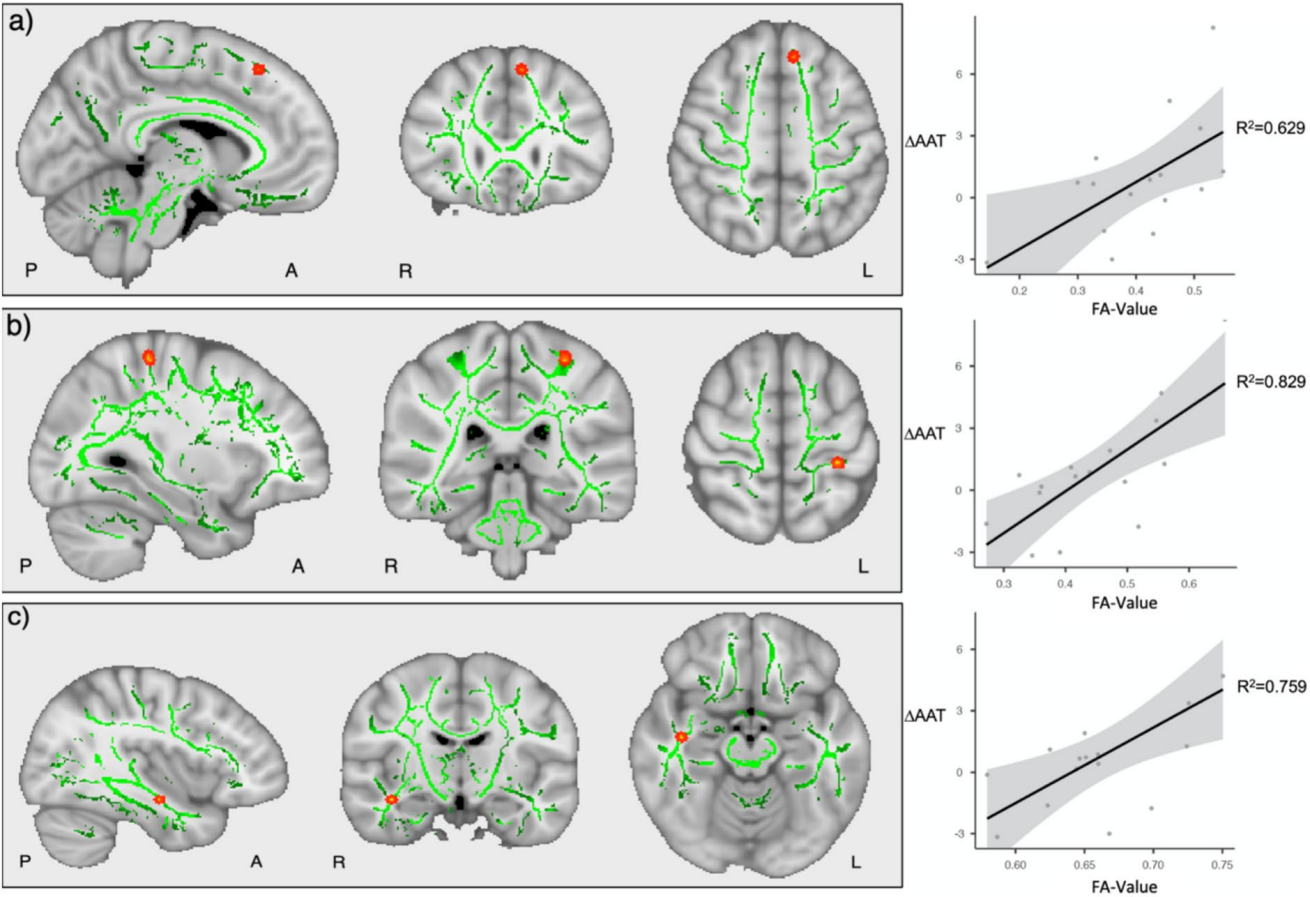
Results of the TBSS-analysis; red-yellow indicates significant clusters, positively correlated with ΔAAT (highlighted with tbss_fill) in **a**) left frontal and **b**) parietal white matter as well as **c**) right temporal white matter. TFCE-based, uncorrected, *p* < .001. FA-mean skeleton (green), overlaid onto MNI152 standard space brain, depicted in radiological convention (*R*   right), *P*  posterior. The adjacent scatterplots show the linear regression of each patient’s individual FA-value (main voxels) with his individual therapy outcome (ΔAAT)

Analysis with TBSS (TFCE-based, uncorrected, *p* < 0.001) showed a positive correlation between FA and ΔAAT in three white-matter clusters in left frontal, left parietal and right temporal structures (Fig. 3; Table 3). FWER control, however, eliminated all significant clusters (*p* > 0.05). The sensitivity analysis indicated a minimum detectable correlation of *r* = 0.53; the design was thus sensitive only to large associations, and smaller effects may have remained undetected. Recomputed without patient No. 17 (early subacute stage), the value was virtually unchanged (*r* = 0.55), indicating that this patient did not skew the group statistical results.

**Table 3:** Significant clusters/FA-values (DTI) and fMRI activity clusters, modulated by ΔAAT

DTI analysis							
Main voxel coordinate (x y z; mm)	Localization		Cluster-size (no. of voxels)	FA-values (patient group)			
	Hemi-sphere	Structure		Mean	SD	Min	Max
–7 28 52	Left	Frontal white matter	28	0.407	0.105	0.144	0.550
–30 –33 59	Left	Parietal white matter	19	0.445	0.104	0.272	0.658
40 –12 –16	Right	Temporal white matter	12	0.664	0.051	0.579	0.750

fMRI analysis						
	Main Voxel activation			Cluster activation		
Contrast	Hemi-sphere	Coordinate (x y z; mm)	*Z*-value	Hemi-sphere	Struc-ture	size (volume mm^3^)
Meaningful language Cluster 1	left	–6 58 24	3.91	bilateral	SFG ACC	4320
Meaningful language Cluster 2	right	56 –26 –20	3.28	right	ITG MTG	2392
Semantics	right	16 –78 –2	3.56	right	LING FUS LOC	4528

*SD*  standard deviation, *SFG*  superior frontal gyrus, *ACC*   anterior cingular cortex, *ITG/MTG*   inferior/middle temporal gyrus, *LING*   lingual gyrus, *FUS*   fusiform gyrus, *LOC*   lateral occipital cortex

Subsequently, FA-values were compared with each other using the Wilcoxon-signed-rank test (*p* < 0.05; one-sided). A significant difference between FA in the right temporal cluster compared to FA-values in both left frontal and parietal cluster was revealed (*p* < 0.001 in each analysis). FA-values in parietal and frontal clusters did not deviate significantly from each other (*p* = 0.105).

### Functional imaging

With respect to the regressor “treatment gain in ΔAAT”, contrasts “Meaningful language” and “Semantics” evoked bilateral and right-hemisphere brain activity patterns, while the other contrasts did not lead to significant functional activation (Table 3).

#### Contrast “Meaningful language”

**Fig. 4:**
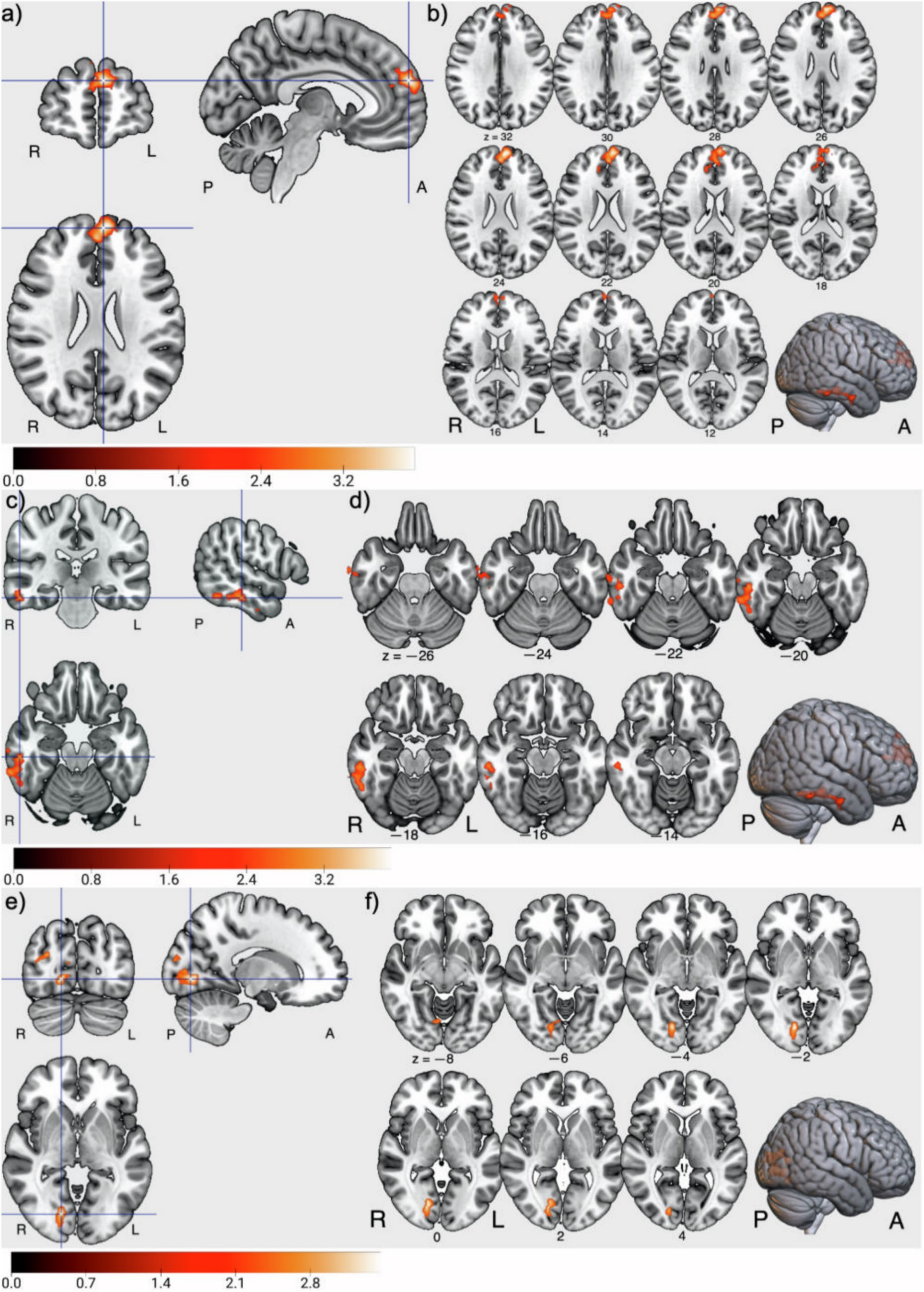
Functional activity in contrasts “Meaningful Language”, Cluster 1 **a**) main voxel, **b**) complete cluster); Cluster 2 **c**) main voxel, **d**) complete cluster) and “Semantics” **e**) main voxel, **f**) complete cluster). All positively correlated with ΔAAT, overlaid on MNI152 standard space brain in radiological convention (*R*  right; *P*   posterior). Z-statistics with Z-threshold 2.3 and Cluster significance threshold *p* < .05. Lighter colors indicate more activation intensity, expressed in t-values

Two clusters of functional activity were observed: in Cluster 1, superior frontal gyrus (SFG) and adjacent anterior cingulate cortex (ACC) activated bilaterally, while the main voxel was located in left SFG (*p* = 0.0003; Fig. 4 a and b). Cluster 2 was located mainly in the right ITG (main voxel, *p* = 0.022), extending into the right MTG (Fig. 4 c and d).

#### Contrast “Semantics”

Functional activity occurred in the right lingual gyrus (LING; main voxel: *p* = 0.0034). The whole cluster extended into the lateral occipital cortex (LOC) and, to a lesser extent, the occipital fusiform gyrus (FUS; Fig. 4 e and f).

#### Analysis in relation to aphasia severity

For lesion volume, significant negative correlations occurred between aphasia severity pre-therapy and lesion volume in INS (*p* < 0.001) and PUT (*p* = 0.044), showing an increase of aphasia severity in relation to greater lesion extent. Concerning white matter structures, significant negative correlations occurred between white matter tract damage in SLF (*p* = 0.006), AF (*p* = 0.003), MdLF (*p* = 0.031), IFOF (*p* = 0.035) and EmC (*p* = 0.001) and aphasia severity prior to treatment. Global lesion size, lesion size to cortical structures, THA and CAU as well as damage to fibers in UF and ILF did not correlate significantly with pre-testing level MPH (Table 2).

Neither DTI nor fMRI measures revealed significant clusters in relation to aphasia severity before treatment.

## Discussion

This study aimed to identify preliminary structural and functional factors that might underlie ILT success. This was examined using a range of structural and functional neuroimaging measures. Lesion volume analysis—both global and specific to cortical, subcortical, and white matter structures—did not correlate with the degree of improvement in language abilities. Consistent with previous literature, the potential for recovery in our patient group was independent of lesion size [26,27]. This finding highlights the capacity for language improvement even in patients with extensive left-hemisphere lesions. In contrast, control analyses revealed a correlation between lesion size in specific subcortical areas (INS, PUT) and white matter tracts (AF, SLF, EmC, IFOF, MdLF) with aphasia severity prior to therapy, as reported before [4, 9]. This underscores that factors influencing aphasia severity do not necessarily determine therapy success. As suggested by other researchers, therapy success appears to be more closely related to factors such as structural neuroplasticity and functional reorganization [14, 27, 50].

In our patient group, ILT success was associated with both white matter structural integrity and functional activity. Regarding white matter integrity, two significant clusters were identified in left frontal and parietal white matter regions that are not typically linked to language processing. Of note, the identified clusters occurred in the uncorrected analysis only and did not survive correction for multiple comparisons. In addition, our method does not allow for the tracing of connectivity routes. Lastly, the sensitivity analysis indicated that the study’s design was sensitive only to large effects. Therefore, we wish to discuss these results with caution. Previous studies have reported correlations between structural integrity in language-relevant fiber tracts, such as the AF and ILF, and modality-specific language improvement [9,10], which was not replicated in our study. This may suggest that improvements in specific language modalities rely on different mechanisms than global language change. While modality-specific improvements might be associated with the structural integrity of brain regions directly involved in that modality, our results suggest that global language profile improvements could be influenced by compensatory mechanisms outside the classical language network, as has been proposed by others [27, 50]. However, more research is needed to test this hypothesis. In a third cluster, structural integrity in the right temporal lobe correlated with ILT success. The right temporal lobe has been associated with healthy language processing [21,22]. Moreover, a growing body of research has demonstrated the relevance of right hemisphere white matter structures to both aphasia therapy outcomes and symptom severity [51]. Given its role in healthy language processing, it seems reasonable to assume that the right temporal lobe contributes to ILT success. Notably, FA values in the temporal lobe were significantly higher than those in the left frontal and parietal clusters. Current literature suggests that particularly intensive recruitment of specific brain structures leads to an increase in FA values in these regions [9, 11]. This may indicate that right temporal lobe structures “work harder” to compensate for homotopic lesions [52]. However, while this finding indicates structural integrity pre-therapy, it remains unclear whether high FA values in the right temporal lobe were already present premorbidly, or if they reflect recovery processes that occurred after the stroke but before study-related DTI measures. In summary, the preliminary findings of our DTI analysis hint towards a possible relevance of structural integrity in brain areas outside the classical language network as well as in the right temporal lobe. Again, given the low statistical threshold in a small sample size, these results should primarily be considered hypothesis-generating for future research.

In addition to the DTI results, the relevance of right temporal lobe structures was also evident in the fMRI findings, which showed right ITG and MTG activity correlating with positive treatment outcomes, as reported in previous studies [14, 53]. While some studies ascribed right-hemispheric activity to compensatory processes of lesion-homologue areas [54], this assumption has been questioned by other authors [23]^.^ This is due in particular to relatively consistent findings of bilateral (albeit left-dominant) temporal activity during healthy language processing, especially in semantic processing tasks such as concept retrieval or sentence comprehension [55,56]. Recent findings have corroborated these results, showing right-hemispheric upregulation of the MTG in patients with chronic aphasia during the course of recovery without a treatment protocol [23]. Consistent with the role of right temporal lobe activity in healthy language processing following recent literature, we propose that our results reflect the recruitment of intact regions within bilaterally organized language network areas, which helps facilitate language improvement. Our combined data suggest that the usage of right temporal lobe structures in our patient group is not only reflected in functional activity, but may also underly the increase of white matter FA in comparison to the other DTI clusters, hinting towards possible mechanisms of Hebbian plasticity [57]. However, as establishing connections between structural and functional parameters with structural neuroplasticity has proven difficult [57], more studies using multimodal imaging techniques are necessary to research possible relationships between structural, functional and behavioral data.

In addition, the functional activity results need to be considered in relation to the fMRI task of passive listening. While bilateral temporal lobe recruitment has been described for language processing, language production tasks are largely left-lateralized [21]. However, we assume that right-temporal activity does not merely reflect the presented fMRI task in our study, since right-temporal functional activity occurred exclusively in relation to therapy success. In contrast, if right-temporal activity were based solely on task content, we would have expected comparable activity patterns in the control analysis, in which the same fMRI paradigm was presented. However, no functional activity was observed in relation to pre-therapy aphasia severity, leading us to assume that right-hemisphere temporal lobe activity is a “real” effect in relation to treatment-induced language improvement.

Further fMRI results revealed activity in bilateral prefrontal and right occipital regions in association with positive therapy outcome, both of which cannot be directly attributed to healthy language processing. Regarding prefrontal activity, SFG/ACC have been associated with the recruitment of domain-general cognitive control networks. These are relevant for a target-oriented execution of tasks without being easily distracted, and their activation has been linked to spontaneous and therapy-induced language improvement [58–60]. Moreover, SFG/ACC activation has been observed in healthy participants during language tasks requiring compensation for increased task difficulty [59, 61]. Structures in the right occipital lobe (LING, LOC, and FUS), on the other hand, are essential for visual processing and mental imagery [62]. However, co-activation in these regions during language tasks has been reported in healthy speakers [63], and studies have found a connection between language task performance and activity in these areas [64]. In addition, activity in right-hemispheric LOC has been described in connection with successful language treatment [65]. The positive influence of activity in these areas may be due to the retention of non-linguistic semantic visual features, which could prove helpful for successfully completing more difficult language tasks in healthy participants. Likewise, aphasic patients with a strong ability to maintain and visualize mental images may be particularly responsive to an ILT program, possibly due to the activation and integration of non-linguistic semantic visual information into language processing, which may help facilitate compensation for linguistic deficits.

In sum, our fMRI findings support the notion that recovery processes are not restricted to a single function i.e. either language or domain-general processes. Rather, we observed activity both in the right temporal lobe, which we attributed to remnants of the language network, and in areas associated with domain-general networks and functions. In line with other research [23, 65], we propose that the interaction between language and cognitive networks may have been particularly beneficial for the successful completion of the ILT program in our patient group.

Our study is limited by the small sample size, which may have affected our results. This was particularly impactful for our DTI results, because it led to the application of a lower statistical threshold. As indicated by the sensitivity analysis, the design was sensitive only to large effects and was therefore underpowered for smaller, more subtle associations. Additionally, there was one patient in the early subacute phase of aphasia recovery, while the other patients were in the late subacute or chronic phase. However, repeated sensitivity analyses excluding this patient showed a negligible effect, and all analyses were controlled for illness duration. Furthermore, this patient showed significant improvements in AAT-MPH at the individual-case level (Table 1). We therefore hold the view that the inclusion of this patient did not confound the group statistical results. However, recovery processes are influenced by illness duration; therefore, future studies may benefit from examining related research aims more closely across different phases of chronicity.

Our fMRI task sample size was smaller than the sample of the structural analyses, and the results may therefore not be directly comparable. Moreover, all patients were grouped into one sample, regardless of their individual therapy outcome. There may have been absent or nonspecific activity patterns in the patients without treatment gain, which might have influenced or even masked significant results in the group analysis. For future studies, examining groups of responders vs. nonresponders to aphasia therapy, as well as including a control group receiving an alternative therapeutic intervention may prove helpful in increasing statistical power and isolating ILT-specific functional activation. This study may thus inspire future multi-centre studies allowing the detection of more subtle effects with even larger clincial sample sizes.

## Conclusion

In our preliminary study, we found first indications, that both structural integrity and functional activity may influence ILT success, whereas lesion size determines initial symptom severity but not therapy-induced language improvement. Notably, both DTI and fMRI analyses highlighted the relevance of the right temporal lobe, which is associated with healthy language processing. This suggests that successful language therapy requires the recruitment of premorbidly relevant language areas, indicating however that not only left hemisphere or perilesional areas may play a role. Additionally, fMRI analysis revealed activity in regions outside the classical language network, specifically areas involved in domain-general cognitive control and non-linguistic semantic visual processing. Our data hint towards a possible recruitment of these areas as compensatory mechanisms for the lesioned language network. Given the exploratory nature of our study, along with the lack of literature in this field of research, we consider our results to be important first pointers for future hypothesis-driven research.

## Data Availability

The anonymized data supporting the findings of this study are available from the corresponding author, upon reasonable request.
